# Physical activity and cognition in the elderly: A review

**DOI:** 10.1590/S1980-57642009DN30300005

**Published:** 2009

**Authors:** Alexandre Leopold Busse, Gislaine Gil, José Maria Santarém, Wilson Jacob Filho

**Affiliations:** 1 Assistant Physician at the Geriatric Service of the Department of Clinical Medicine of HCFMUSP, PhD in Science from FMUSP, São Paulo, SP, Brazil.; 2 Neuropsychologist, post-graduate qualified at the Institute of Psychiatry of HCFMUSP, coordinator of the Memory Stimulus Center of the Alemão Oswaldo Cruz Hospital and of cognitive assessment for Fleury laboratories, São Paulo, SP, Brazil.; 3 Coordinator of the CECAFI – Center for Studies in Physical Activity Sciences of the Geriatrics Discipline of the FMUSP, Medical Doctor from FMUSP, São Paulo, SP, Brazil.; 4 Associate Professor of the Geriatrics Discipline of the University of São Paulo School of Medicine, São Paulo, SP, Brazil.

**Keywords:** Aging, dementia, cognition disorders, motor activity

## Abstract

Physical activity has been indicated as a strategy to promote health in the elderly, as well as to encourage the maintenance of functional capacity, and acts in the prevention and control of various diseases. In recent years, there has been great interest in studying the benefits of physical activity in the preservation or even improvement of cognitive performance in both the elderly without cognitive impairment and in elderly patients with some degree of cognitive impairment or dementia. The majority of epidemiological studies and clinical trials have evaluated aerobic exercises while few have assessed resistance exercise programs. The objective of this review was to examine the effects of different types of physical activity on cognitive function of elderly individuals with or without prior impairment.

## Abstract (pt)

A atividade física tem sido amplamente indicada como uma estratégia para a promoção de saúde no idoso, pois além de favorecer a manutenção da capacidade funcional, atua na prevenção e controle de diversas doenças. Nos últimos anos, tem havido um forte interesse em estudar os benefícios da atividade física na preservação ou mesmo na melhora do desempenho cognitivo tanto em idosos sem comprometimento cognitivo como em idosos com algum comprometimento cognitivo e demência. Os estudos epidemiológicos e a maioria dos ensaios clínicos avaliaram os exercícios aeróbios e poucos ensaios clínicos adotaram programas de exercícios resistidos. O objetivo desta revisão é analisar os efeitos de diferentes tipos de atividade física na função cognitiva de idosos com ou sem comprometimento prévio.

The MEDLINE and LILACS scientific databases were searched for articles in English or Portuguese published between January 2004 and March 2009 which matched the following DeCS/MeSH key words: motor activity, physical fitness, aerobic, aerobic capacity, physical activity, resistance training, cognitive disorders, memory disorders, dementia, aged, elderly, old.

The most relevant review articles, observational studies and randomized clinical trials were included, along with some studies listed in their bibliographic references.

## Introduction

Physical activity has been widely indicated as a strategy for promoting health in the elderly as well as for maintaining functional capacity, and acts in the prevention and control of a range of diseases including arterial hypertension, cardiovascular events, *diabetes mellitus*, osteoporosis, osteoarthritis, obesity and depression. In recent years, there has been growing interest in studying the potential of physical activity to preserve cognition, and particularly as a protective factor against developing dementia. There has also been great interest in determining the efficacy of exercise for improving cognitive performance both among elderly with dementia and in those with mild cognitive impairment, where in the latter case the effects of exercise on lowering the conversion rate to dementia has also been a focus of study. The aim of this review was to analyze the effects of different types of physical activity on cognitive function in elderly individuals with or without known impairment.

### Performance improvements in elderly without cognitive impairment

A prospective, observational study by Yaffe and cols. involving 5,925 community-dwelling elderly women who were regular walkers and monitored for six to eight years, found poor performance on cognitive tests to be inversely proportional to the distance walked and energy consumed per week during walks.^1^

A meta-analysis conducted by Colcombe and cols. including 18 randomized, longitudinal studies published between 1966 and 2001, concluded that aerobic exercises may yield consistent improvements in cognitive performance of sedentary elderly without dementia. Further analyses identified several moderating factors of the relationship between physical fitness and cognition: among the cognitive functions improved, executive functions appeared to benefit most; the effects of aerobic exercises were enhanced when combined with strength and flexibility training; studies involving a large number of women showed greater benefits than those containing few women.^2^

Van Gelder et al., who followed 295 elderly men born between 1900 and 1920 for more than ten years in the FINE Study, reported a 3.5-fold greater decline in cognition among individuals who walked little compared to those who walked more than 3 miles per day.^3^

Other studies have also suggested that physical activity improves executive functions. An intervention study conducted by Scherder and col. involving light physical exercise in older old showed improvements in tests assessing executive functions, despite a casuistic of only forty three participants.^4^ In an observational study in 120 elderly, Bixby and cols. found a significant correlation between better performance on the Stroop test and higher levels of physical activity.^5^

The Cochrane Foundation undertook a systematic review which assessed the effect of aerobic physical exercise on cognitive function in older people without known cognitive impairment. Eleven randomized, controlled studies in individuals aged 55 years or older were selected. Evidence was found suggesting that aerobic physical exercise improved cognitive function in elderly persons, with benefits seen in motor function, cognitive speed and attention. However, these findings were insufficient to prove that the improvements observed in cognitive function were in fact due to improved cardiovascular fitness, although temporal association suggested such a link. Further studies are needed to confirm whether such benefits are specific to aerobic training or may be derived from any type of physical exercise.^6^

### Lower dementia incidence

A meta-analysis conducted by Heyn and cols. which included 12 studies performed between 1970 and 2003, assessed whether exercise training improved cognition among elderly persons with dementia and cognitive impairment. The predominant intervention type was walking where this produced moderate results in terms of efficacy of the physical exercises in improving cognition.^7^ One of the studies included was that by Laurin and cols. which followed nine thousand elderly persons for a five year period and showed a 40% lower risk for cognitive impairment and 50% lower risk for developing Alzheimer’s disease in individuals who walked three times per week or more.^8^ The review by Rockwood and col. of 22 observational studies, each having a follow-up period of five years or more, compared physically active individuals versus sedentary individuals and demonstrated that physical activity reduced the relative risk of dementia, cognitive impairment and cognitive decline.^9^ Another review carried out by Taaffe and cols involving more than 2,200 Japanese-American elderly men from the Honolulu-Asia Aging Study, without dementia and aged between 71 and 93 years, found that after a mean follow up of 6.1 years, the risk for developing dementia was reduced by half in the group with a higher level of physical activity compared to the group with a lower level.^10^ Larson and cols. conducted a clinical trial in 1,740 elderly persons and concluded that doing regular aerobic exercise was associated with a reduced risk of Alzheimer’s disease.^11^

Lautenschlager and cols. conducted a study which sought to demonstrate that physical exercise improves cognitive function in older adults with mild cognitive impairment. The trial involved a cohort of 170 elderly persons and the experimental group began a 24-week physical exercise program (home-based) which resulted in a weekly increase in physical activity of 142 minutes compared to the control group. Overall cognition was assessed using the ADAS-cog and other specific tests for episodic memory, attention and working memory. Instruments assessing quality of life and depression were also applied. The only test which revealed a significant difference was the ADAS-Cog which showed improvement in the intervention group six months after program commencement, where these improvements remained for at least 12 months after training.^12^

### Studies in demented patients

Clinical studies in patients with dementias are rare. A recent systematic review by the Cochrane Foundation included only two large randomized studies, whose findings suggested insufficient evidence to affirm the efficacy of physical training in maintaining or improving cognition, functionality, behavior, depression and mortality among demented elderly.^13^

### Resistance exercises and cognition in elders

The effects of resistance exercises on cognition have not been widely investigated. A study by Perrig-Chiello in 46 elderly volunteers assessed an 8-week resistance training program and found no significant effect on memory among participants.^14^ Lachman and cols. submitted 210 community-dwelling older adults to a 6-month home-based strength training program and observed no difference in memory versus controls. However, the participants who progressed most on weights during the training obtained significant improvements in working memory.^15^

In a randomized clinical trial by Cassilhas and cols. in 62 elderly without cognitive compromise, a significant improvement in both working and episodic memories were found in the group undergoing resistance training for six months.^16^ Another randomized clinical trial in 74 older adults carried out by Liu-Ambrose and cols. showed that a combined home-based strength and balance training called the Otago Exercise Program, significantly improved executive functioning after 6 months in older adults aged 70 years or more with a recent history of falls. The conclusion of the study challenged the hypothesis that cognitive and neural benefits of physical activities occur within the setting of social commitment to perform exercise.^17^

A randomized controlled trial by Busse and cols. in 32 elderly individuals with memory impairment showed a significant improvement on episodic memory tests in the group undergoing resistance training exercises for nine months.^18^

### Structural and functional correlations

A study involving 165 non-demented elderly aged between 59 and 81 years, adjusted for age, gender and level of schooling, found strong correlation between increased aerobic fitness and greater hippocampal volume bilaterally on nuclear magnetic resonance imaging. Although studies in rodents have shown that exercise increases hippocampus size and spatial memory, this was the first study to show this effect in humans. A significant association was also found between aerobic fitness and performance on memory tests. Erickson and cols. therefore concluded that increased levels of aerobic fitness may be associated with increased hippocampal volume in elderly individuals, which can lead to improved memory performance.^19^ Colcombe and cols. conducted a randomized study involving a group of walkers who exercised for three times per week and a control group. All participants performed selective attention tests during the functional magnetic resonance protocol. After six months, the walking group performed better on tests and exhibited increased activity in frontal and parietal regions involved in efficient attention control.^20^

A study of more than 2,200 Japanese-American men between the ages of 71 and 93 has found that elderly men who are sedentary or walk less than a quarter of a mile per day are nearly twice as likely to develop dementia and Alzheimer’s disease compared to men who walk more than two miles per day.^10^

### Possible molecular mechanisms

The mechanisms underlying the protective effect of physical activity against the development of Alzheimer’s disease and other types of dementia, as well as the improvements in cognition of healthy individuals, remain unknown. Improvements in cerebral perfusion and cardiovascular performance have been proposed.^21^

Studies in animals have yielded information on the effects of exercise not obtained in humans. Exercise appears to increase neuronal plasticity in both old and young animals, due to increased levels of RNAm and BDNF neurotrophin (Brain-derived neurotrophic factor). These chemical mediators may contribute to the induction of neurogenesis in the dentate gyrus influencing the formation of new neural networks.^22^

## Discussion

There is an increasing body of evidence pointing to the beneficial effects of physical activity on various cognitive functions.^23^ Studies show that physical activity improves executive functions,^2,4,5^ attention,^6,20^ cognitive speed,^6^ working memory^15,16^ and episodic memory.^16,18^ A review by the Cochrane Foundation found evidence that aerobic activity is beneficial to cognitive function in older people without known cognitive impairment.^6^

Observational studies^7,9,10^ and a clinical trial^11^ have associated moderate physical activity with reduced risk for developing Alzheimer’s disease. However, although another study, by Lautenschlager and cols., demonstrated that physical activity improved cognitive function in older adults with mild cognitive impairment, a group at risk for evolving to dementia, no reduction in conversion to dementia was observed after 18 months’ follow up.^12^ Further investigation is warranted to ascertain whether physical activity reduces conversion to dementia, particularly in older adults with Mild Cognitive Impairment.

According to the systemic review by Cochrane Foundation,^13^ there is currently insufficient evidence regarding the efficacy of physical activity training among individuals with dementia. Further studies are therefore needed to identify not only the effects of physical activity on cognition of demented persons, but also its impact on the quality of life of their caregivers and family members.

Clinical trials investigating the effects of strength exercises on cognition are scarce, while available studies tend to involve few participants and have short follow ups. Investigating elderly with no known cognitive impairment, both the studies of Cassilhas and cols. and Liu-Ambrose and cols. showed an association between strength exercises and improved cognitive performance.^16,17^ The study by Busse and cols. showed similar benefits in elderly individuals with known memory impairment.^18^ The systematic review by the Cochrane Foundation concluded that the evidence was insufficient to clarify whether improvements in cognitive function following aerobic exercises stemmed from improved cardiovascular fitness or whether benefits can be derived from any type of physical exercise^6^. The review by Calcombe and cols. concluded that the effects of aerobic exercises were enhanced when combined with strength training.^2^ These findings corroborate the guidelines by the American College of Sports Medicine^24^ and the American Heart Association^25^ which recommend a mix of resistance and aerobic exercises to promote good health, only now with some evidence of effects on cognitive health.^17^

## Conclusions

There is now substantial evidence that physical activity is beneficial to cognitive health, although the molecular mechanisms involved remain unknown.

Aerobic activity also benefits cognitive function in elderly persons with no known cognitive impairment. However, the evidence is insufficient to clarify whether cognitive improvements are due to improved cardiovascular fitness or whether these benefits can be derived from any type of physical exercise.

Few studies have investigated resistance exercises, although there is evidence of cognitive improvement in the studies performed to date. Moreover, the effects of aerobic exercise on cognition were shown to be enhanced by combining it with strength training.

In addition, there is some evidence that physical activity lowers the risk of developing Alzheimer’s disease, but further randomized trials are needed to confirm this theory.

The evidence currently available regarding the effectiveness of physical activity training for improving cognition, functionality and behavior in dementia sufferers is insufficient. Moreover, future randomized studies should also assess the quality of life of caregivers and the need for hospitalization.
